# Ten simple rules for turning your qualifying exam into an NIH-style fellowship proposal: A guide for graduate students

**DOI:** 10.1371/journal.pcbi.1014420

**Published:** 2026-06-18

**Authors:** Courtney Peña-Lima, Cameron S. Bader, Brendan K. Ball, Troy C. Dildine, Mekhala V. Dissanayake, Iris van ‘t Erve, Albina Ibrayeva, Amy Nippert, M.K. Quinn, Chelse Spinner, Samuel Thompson, Antonio Tomasso, Crystal M. Botham

**Affiliations:** 1 Biosciences Grant Writing Academy, Stanford University, Stanford, California, United States of America; 2 Division of Blood and Marrow Transplantation and Cell Therapy, Stanford University School of Medicine, Stanford, California, United States of America; 3 Department of Pathology, Stanford University School of Medicine, Stanford, California, United States of America; 4 Department of Anesthesiology, Perioperative, and Pain Medicine, Stanford University School of Medicine, Palo Alto, California, United States of America; 5 Department of Pediatrics, Stanford University School of Medicine, Stanford, California, United States of America; 6 Stanford Cancer Institute, Stanford University, Stanford, California, United States of America; 7 Department of Radiation Oncology, Stanford University, Stanford, California, United States of America; 8 Department of Neurology and Neurological Sciences, Stanford University School of Medicine, Stanford, California, United States of America; 9 Department of Genetics, Stanford University School of Medicine, Stanford, California, United States of America; 10 Hagey Laboratory for Pediatric Regenerative Medicine, Department of Surgery, Stanford University School of Medicine, Stanford, California, United States of America; Carnegie Mellon University, UNITED STATES OF AMERICA

## Abstract

Qualifying exams, often referred to as “quals” or candidacy exams, are an important milestone in doctoral programs. Although the style of quals varies greatly by program and institution, it is usually a proposal that requires students to develop research ideas as well as their scientific writing skills. Many quals are modeled after funding mechanisms that graduate students can apply to and on a topic that the student will pursue in their dissertation. This paper offers graduate students a step-by-step guide on how to turn their quals into a fellowship-style research proposal, using National Institutes of Health (NIH) mechanisms as a benchmark, as this is the norm within US research institutions. This paper will be most useful for students who have completed or are in the process of completing proposal-based qualifying exams, usually in the second year of a doctoral program.

## Introduction

Graduate school is a time of transformation as you develop from being a consumer of science to a producer of science. In the US, many PhD programs require a capstone research project like a qualifying exam (“quals”) where students develop research ideas and writing skills. In preparing for your qualifying exam, you’ve invested a lot of time into writing about your research. Did you know you can turn your qualifying exam into a grant proposal or fellowship to fund your graduate career? Graduate students can apply to a variety of external and internal funding opportunities.

Writing research proposals is a way to put your future as a scientist in focus as a graduate student. Funding opportunities for graduate students (often referred to as fellowships, such as US National Institutes of Health (NIH) fellowships) typically require three interwoven plans: research, mentoring, and career development. It is also a great way to build your resume as securing funding has benefits for many professions including as an independent researcher [1]. Even if not funded, the act of writing a proposal is a valuable skill to develop as it strengthens your critical thinking and writing skills [2]. Writing a proposal enhances your science and career development as it requires you to engage with your research in new ways, think more deeply about future career plans, and identify the type of training and support you will need to reach your goals. It demonstrates your commitment to your research and solidifies relationships with mentors by seeking their feedback. With external funding, you have additional agency to pursue your own research interests as well as use available funds for professional development training, like attending conferences. By applying for external funding and familiarizing yourself with the process, you are gaining valuable knowledge that will be beneficial to your development as a researcher in the long-run, so think of this experience as a journey and not an outcome.

While the process of applying can be time-consuming, it is well worth the effort because it is an opportunity to develop your skills as a scientist. In the rules below, we have laid out a step-by-step guide to turn your qualifying exam into a fellowship-style research proposal. These rules will be most useful if you have already completed your quals or will in the near future. While they are tailored to US funding mechanisms, the concepts of the rules may be applicable to other contexts.

## Rule 1: Identify a funding opportunity that fits your graduate program’s timeline

The first step to getting funded is understanding the funding landscape, including funders’ deadlines, eligibility, and interests. Many universities compile lists of funding opportunities relevant for their graduate students, such as Stanford University’s Funding Train [3], UCLA’s GRAPES [4], Harvard University’s CARAT [5], and John Hopkins’ funding list [6]. Check to see if your university has something similar. External funding may include the trainee focused NIH Ruth L. Kirschstein National Research Service Award (NRSA) Fellowship or the American Heart Association Predoctoral Fellowship. Make a comprehensive list of potential funding opportunities, including relatively soon deadlines (6–9 months) as well as future opportunities (1+ years). Add to your list any relevant internal funding opportunities as well, such as seed funding or a slot on an institutional training grant, such as NIH’s T32.

Deadlines and eligibility are frequently strict (e.g., citizenship status, time in program), but many funders have broad interests and cast a wide net for research that might someday impact their stated interests. Your goal is to understand the funder’s values and priorities so you can imagine how your work can align with their interests. Carefully read their website, announcements, and see who they have funded; if possible, talk to past awardees. For the NIH, the RePORTER Matchmaker [7] allows you to search key terms in your application to identify similar funded research. This process can also take time because the process of identifying a relevant funder might not be immediately obvious or straightforward and you may need to adapt your work to match the funding call.

Once you have identified an opportunity (or opportunities), reach out to relevant staff to check alignment, such as NIH’s Program Officers [8]. When the data are public (e.g., NIH Data Book [9]), look at both the percentage and total number of awards given. For NIH, each institute (for example National Heart, Lung, and Blood Institute) sets its own priorities for funding, meaning proposal success rates can vary widely and you may want to tailor your proposal to the institute that funds more fellowships each year.

## Rule 2: Create a checklist and timeline for submission

Once you’ve identified a potential funder, the next step is to make a “checklist” or list of the documents required for your proposal. Carefully read the proposal’s instruction guide, which will describe the documents you need to prepare. Some funders will also share the review criteria that reviewers will use to rank proposals, which you should use to inform your writing as well. Gather funded and even unfunded sample proposals from peers or other repositories, such as Open Grants [10], to examine how other grant writers approached their proposals; for tips on how to examine funded proposals, read: How to Gain a Competitive Edge in Grant Writing [11]. Once you have gathered all the relevant information, draft a checklist with all the necessary steps needed to submit your proposal. Do include major proposal documents like the research (Rule 5), mentoring (Rule 6), and career development (Rule 7) plans, internal requirements (Rule 9), required letters (Rule 6), facilities documents, and other resources or equipment. Other documents may be optional, or only required when certain types of methods, such as when vertebrate animals or human subjects are proposed. It may be helpful to group the documents on your checklist into categories such as core documents (requiring lots of iterative feedback (Rule 9), e.g., the research plan), letters or supporting documents (requiring documentation from others, e.g., a letter from your mentor), and standard content (these are commonly referred to as boiler documents) that may be available through your mentor which require minimal changes (e.g., list of equipment available for you in the lab).

Now that you are familiar with the specific documents you will need, figure out *when* you’re going to complete them. We recommend between 3 and 6 months before the deadline to prepare a compelling proposal. Your timeline should provide time for outlining, drafting, seeking feedback, and revising. Remember that some universities will require review of the final proposal too (Rule 9). Build into your timeline plenty of buffer time (20–30%) to accommodate unexpected situations and other major commitments. Also, add breaks into your timeline so you can return to your proposal with fresh eyes. Consider making your timeline visually appealing and keeping a copy at your writing station so you are reminded of your daily (or weekly) writing goals.

Equipped with both your checklist and timeline, create a tracking system that will work for you in documenting your process and highlighting remaining tasks because writing a proposal requires stellar project management skills.

It is important to note that, as a graduate student, you are still developing many of the skills required to write a proposal and may experience a miscalculation in how long it takes to complete certain writing tasks. Know that this is part of the learning process and may require you to adjust your timeline as you re-align your estimations to reflect your actual writing time.

## Rule 3: Get your mentor on board

Grant writing is rarely a solo effort. Turning your qualifying exam into a compelling proposal requires planning, communication, and community.

### Start with self-reflection

Identify your strengths and weaknesses as both a researcher and a writer. Are you strong in research planning but less practiced in articulating the story behind it? Do you thrive on frequent feedback or prefer to polish independently? Knowing this helps you and your mentor tailor support and allocate time effectively.

### Engage your mentor early and intentionally

Preferably, while you’re writing your qualifying exam, share your plan to convert it into a proposal with your mentor and discuss how this aligns with your long-term goals. The primary mentor and any co-mentor(s) are an important part of your proposal too. Clarify expectations for meeting frequency, draft readiness, and feedback timelines, see example Mentor-Mentee Planning Guide [12]. Treat your mentor as a collaborator rather than an evaluator, i.e., someone who helps shape the proposal’s vision, not only its details. Follow through on sending drafts to your mentor to allow enough time for your mentor to provide thoughtful feedback. If your mentor’s availability is limited, communicate your needs early and identify other mentors or senior peers (e.g., postdocs) who can provide targeted feedback. Specific questions to discuss with your mentor are:

- At what stage(s) of the proposal writing process (brainstorming, outlining, providing feedback on drafts, proofreading, etc.) will the mentor be most involved?
- What parts of the proposal would the mentor like to review first?
- How often, and in what forums, should you update your mentor on your proposal writing progress and/or challenges?
- How far in advance should you share drafts for feedback?
- Are there collaborators, prior awardees, or administrative contacts the mentor recommends you reach out to?

Note that you will need a broad network of mentor support, aside from your main mentor, which is addressed in Rule 6.

### Stay accountable

Set recurring check-ins with your mentor to maintain momentum. Regular meetings will reinforce progress and result in a stronger proposal.

## Rule 4: Set yourself up for writing success

Writing is an important part of being a scientist [13,14]. Developing strategies to counter barriers to writing progress, such as procrastinating or hitting writer’s block, is a critical component to writing productivity and future success as a scientist. There are specific resources on overcoming writing resistance [15–17], and we have identified a few methods to address these barriers, detailed below. It is important to evaluate your writing practice often, and when you are feeling your writing productivity is lagging, try a new method.

### Internal accountability

First, it is important to build time for writing within your daily schedule, same as you would do for your lab work or experiments. As a graduate student you are likely still figuring out your writing routine. For some students, 30-min chunks throughout the week is a functional and manageable writing goal, for others 2-hour blocks are ideal. You can learn what works for you through self-assessment and experimenting with different time allotments and move forward accordingly. Second, if you are experiencing procrastination or dread at the thought of writing, pause and participate in either a guided meditation or slow breathing. Being able to activate your parasympathetic system to slow down and relax will help you to better engage with the sources of your feelings. Third, we advocate for a “just-write” approach. It is quite common for scientists and writers, regardless of experience, to have moments of low motivation. It is through the process of continual writing that you can bypass those feelings and increase motivation. Additionally, starting with a free-write or brainstorming exercise may help you to process and release some of these negative emotions. Beyond a just-write approach, consider trying the Pomodoro Method of multiple rounds of writing with intermittent breaks [18].

### External accountability

A key tool that can increase writing progress is writing in a group or social structure. Availability for this option can vary, but could include writing in pairs, small groups, or in a classroom setting. The social space may increase specific social pressures, but it is often positive and can be harnessed to promote constructive outcomes such as: increased motivation, increased resilience and focus, and ultimately improved performance. Be sure to be selective in your writing group by including people who can support you in your writing and provide a source of encouragement. The proper environment and group setting, built with the right intentions and organization, can further promote success.

## Rule 5: Revisit the research strategy

You likely received feedback on your qualifying exam documents already, which is a great starting point for drafting your research proposal. The research plan for graduate funding opportunities usually consists of two sections: 1) Specific Aims and 2) Research Strategy. The specific aims section or document provides the executive summary of the research proposal and the research strategy, consisting of several subsections like the significance, innovation, approach, timeline, and future directions, provides the details of what you will do. Always read instructions carefully because funders can have different requirements and page limits. For example, NIH NRSA Fellowships do not require an innovation subsection.

To begin, revise (or draft anew) an outline of your specific aims, we recommend reviewing The Anatomy of a Specific Aims Page [19] and Writing Your Specific Aims [20]. Remember that you may need to adjust the scope of your qualifying exam proposal to fit the requirements of your targeted funding opportunity. After drafting your specific aims outline, seek feedback from your peers and mentors (Rule 8). Next, begin drafting your specific aims document using complete sentences and paragraphs. Again, seek feedback from peers and mentors as well as revise to increase clarity, conciseness, and compellingness. Finally, draft the research strategy subsections. Ask yourself these questions to help structure the research strategy [21]:

- Why is my research needed? (Significance)
- What is novel about my research? (Innovation)
- How will I tackle the proposed research? (Approach)
- How long will the research take? (Timeline)
- What is the expected return on investing in this research? (Future directions)

Continue to seek feedback as you outline and draft each subsection of the proposal. You may need to make adjustments to your specific aims as you continue to revise based on feedback.

### A note about data in proposals

Data, presented in the text, figures, or tables, are used to demonstrate feasibility for the proposed research or to support your hypothesis. Intentionally organizing and presenting the data, either published or preliminary, within your research proposal can make all the difference in your chances of being awarded funding.

You may not have much in terms of your own preliminary data, depending on what stage you are in your training. This may feel daunting, but it is still possible to write an effective proposal because you can cite published data or even data from others within your research group. You can also include schematics, such as detailed analysis pipelines or your proposed hypothesis, as figures within your proposal.

When you do have data, it is critical to present them in a logical manner including informing the reviewer exactly what the results mean. Throughout your proposal, contextualize the relevant data and findings and describe why they matter. Articulate how the findings you present demonstrate that your research is feasible and justifies your approach. Equally important, text within your proposal, even those in figures, should be legible and easy to read.

If you are considering collecting additional preliminary data, a question to ask yourself is if you have reasonable time to conduct the experiments or analysis while also writing your proposal. If you do, great! Determine the most critical results or findings crucial for your proposal and get started on those analyses. For example, if your investigation requires significant time for a large-scale cohort study (e.g., mouse study, human samples, etc.), consider planning a pilot study with a small sample size.

## Rule 6: Assemble your mentoring team and draft your mentoring plan

Creating a mentoring plan is a crucial step not only in fostering your professional development and achieving your career growth but also in defining the success of your grant application. A well-structured and personalized mentoring plan provides guidance, support, and valuable insights that uniquely enhances your research capabilities and future successes. The mentoring team consists of your primary mentor, potential co-mentor(s) (if necessary), and advisors (i.e., collaborators, contractors, consultants, committee members). The mentoring plan should clearly outline how the primary mentor will guide your research and career development including the specific skills and experiences you need to prepare for your next career stage. Key components of a mentoring plan include defining goals and milestones, detailing specific career development activities, describing the mentor’s role and expertise, and providing a strong communication plan to ensure a successful mentor-mentee relationship.

When developing the mentoring plan, make sure to distinguish between roles of the primary mentor, co-mentor(s), and advisor(s). The level of responsibility, involvement, and commitment to the research proposal will be different for each role. The primary mentor will provide overall supervision and guide the applicant’s research and career development. A co-mentor is a member of the mentoring team that may bring forth an additional expertise or skillset needed to facilitate the research. An advisor likely contributes specific expertise in the proposed research. The mentor and co-mentor often write the mentor plan (often as a letter to reviewers) that is included within the proposal. You may also include letters of support from advisors, which detail their specific contribution and commitment to supporting you.

### A note about letters of recommendation

Letters of recommendations are required for some proposals too. A letter of recommendation is typically written by a referee (e.g., an individual familiar with your qualifications) but who doesn’t have a role in the research proposal. The letter of recommendation should be tailored to the funding opportunity and include specific examples about the applicant’s qualifications and relevant skills, such as applicant’s ability to conduct rigorous research and their research accomplishments to date [22]. To assist your referees in developing their letter, provide a brag sheet [23] that highlights your strengths and contributions as well as provides relevant talking points for the recommender [24]. Consider individualizing your brag sheets for your recommenders so your recommendations are complimentary and not redundant in the information they provide to reviewers. Together, the mentoring plan and accompanying letters should showcase your potential to become a productive independent researcher and radiate their excitement in augmenting your research and career development.

## Rule 7: Create your career development plan

The career development plan is required for most graduate funding opportunities because funders are as interested in augmenting your development into an impactful scientist as making contributions to science. Because of this important goal, the career development plan should be written early in your proposal writing process to allow time for feedback and alignment with the research and mentoring plans. It is important to show consistency and cohesion across the three plans: research, mentoring, and career development.

Start with a careful assessment of your current skills and the skills needed in order to obtain your career goals and objectives. This exercise is useful for writing your career plan, as well as planning and prioritizing relevant training opportunities throughout your graduate training. In general, focus your career development plan on a few key skills. Prioritize the skills needed for your proposed research as well as professional development skills such as scientific writing, mentorship, and presentation. You don’t want to propose skills or training that you have already completed. Instead, acknowledge you already have foundational skills, but require additional training within specific areas. For example, describe your strong foundation in bioinformatics programming and propose additional coursework in Python. Present this level of background and rationale for each proposed skill. Clearly defining the *why* and *when* for each skill is essential and should align with the proposed research and timeline.

If you are proposing skills that are not already utilized by your lab, propose a co-mentor or advisor (Rule 6) to provide those skills. Clearly identify who will train you for each of the proposed skills.

Your career development plan must follow the Goldilocks principle and be just right in terms of its scope. Proposing too many commitments outside of the proposed research won’t seem feasible to reviewers. While it is fine to spend one semester as a teaching assistant or doing outreach, these time commitments should be balanced such that your primary focus is the proposed research. It is also important to tailor the training to your training stage. For example, early-stage graduate students can include introductory coursework, but later stage graduate students would include more advanced courses.

## Rule 8: Seek feedback

An important feature of the grant writing process is the iterative cycles of receiving feedback, making revisions, and seeking further feedback. You likely already received feedback from your committee and your mentor on your quals. Seeking additional feedback from peers and mentors can reveal gaps in the research’s significance, design, methodology, etc., enabling you to address these issues before submitting your proposal. Additionally, seek feedback on your two other plans (career development and mentoring plans) required for fellowship research proposals. Both peer and faculty feedback are valuable for improving your writing [25], and shown to increase the likelihood of funding success [26].

### Participate in peer review

Peer review in this context refers to feedback and assessment shared reciprocally among co-learners (see [26] for worksheets for an effective peer review process), such as meeting with other graduate students to review each other’s drafts and provide feedback. Feedback from peers is associated with overall improved writing quality compared to receiving feedback from a single expert, because peers provide quantitatively more feedback that is less directive [25,26]. Additional benefits of participating in the peer review are that it provides opportunities to “think like a reviewer,” which can inform your own writing by anticipating reviewer questions and comments.

Don’t be concerned about participating in peer review if you are not an expert in the given proposal’s area. Your feedback will mirror potential reviewers, who may also be unfamiliar with the proposal’s research topic, providing crucial insights during the proposal development process. Getting feedback from researchers outside your research area can identify aspects of your proposal that are unclear, such as jargon that is subject matter specific, that those familiar with your work (e.g., mentors, lab mates) may not notice. Incorporating diverse perspectives will lead to more innovative ideas and approaches that strengthen your proposal’s impact.

### Seek feedback from your mentoring team

Your mentor and members of your mentoring team (co-mentors, advisors) are other important sources of feedback. It is critical to establish expectations regarding sending drafts, receiving feedback, and incorporating revisions early in the grant writing process (Rule 3). Carefully consider what sections (or subsections) of the proposal should be reviewed by members of your mentoring team. For example, it may be helpful to seek targeted feedback on Aim 2 from a specific advisor on your mentoring team because they are an expert on the methods proposed for Aim 2. Ask for different types of feedback during different stages of your writing, for example, early in your writing process ask for feedback on your logic and presentation of ideas rather than on word choices or grammar.

## Rule 9: Submit your proposal

Typically, you do not submit your proposal directly to the funder. Instead, it is submitted by an institutional signing official at your university; each university has its own internal processes for submitting proposals. Typically, universities will review the final proposals a full 5 business days in advance of the funder’s due date. This internal deadline is necessary to allow time for the institutional signing official to review your proposal for completeness and compliance. Thus, it is imperative that you are aware of your university’s internal processes so ask your mentor as well as peers about internal processes. A good rule of thumb is to aim to have your proposal completed at least one week before any internal deadlines (Rule 2), to allow you a bit of breathing room as you address last-minute problems that always arise (i.e., formatting issues, missing documents, etc.).

## Rule 10: Celebrate and follow through on next steps

Submitting a grant requires a celebration, a step that must not be skipped. Grab a cupcake or your favorite treat to acknowledge this momentous milestone. Even better, embrace this triumphant moment with a picnic or dinner date with a friend or your pet.

After celebrating, it is time to follow through on next steps. Some funders allow submission of post-submission materials, which is an opportunity to update your reviewers on progress since the submission of the proposal. Allowable post-submission materials vary by funders, but can include updates on new publications, recent presentations, exciting preliminary data, as well as revisions to the proposal. NIH post-submission materials are requested by the Scientific Review Officer (SRO) via email, about one month before the Scientific Merit Review meeting, so mark your calendar when to expect this request and create a plan for generating a robust post-submission response. Additionally, if your proposed research requires oversight, such as from an Institutional Review Board (IRB) or Institutional Animal Care and Use Committee (IACUC), submit or update the relevant protocol as soon as your proposal is submitted. It is important to ensure you are ready to start your research as the funder may give you only days to complete time-sensitive documents when they are finalizing funding decisions, such as NIH’s Just in Time (JIT) process.

NIH, like some other funders, prohibits applicants from having identical proposals under review at the same time. However, applying to different funding sources, such as federal (e.g., NIH’s F31), foundational (e.g., American Heart Association Predoctoral Fellowship), and internal (e.g., seed funding), is often allowed, but carefully review instructions for information on duplicate submissions and scientific overlap policy. Applying to different funding sources increases your chances of becoming funded, although you may not be able to accept multiple awards. Proposal writing should be a process of continuous progress, like your research. Work towards the next funding opportunity on your list by generating a checklist and timeline (Rule 2). Then, revise your proposal drafts to be responsive to the new review criteria and instructions. You have invested an enormous amount of time and resources already in drafting your proposal so keep moving it forward.

Lastly, most funders will share reviewers’ feedback about your proposal’s strengths and weaknesses. Relish any comments from reviewers’ that highlight your proposal’s strengths. Conversely, negative feedback can invoke strong emotions so allow time for the self-care you need to view the feedback as constructive. As you develop a plan for potential next steps for your proposal drafts, consider if the reviewer’s concern is major or minor. Finally, revise your proposal and resubmit or submit as a new proposal.

## Conclusion

Turning your qualifying exam into a grant proposal may seem daunting at first, but it can become one of the most rewarding parts of your graduate training. Think of proposal writing not as a hurdle, but an opportunity to discover your voice as a researcher and communicate the excitement of your science to the world.
